# Tumour suppressor microRNAs contribute to drug resistance in malignant pleural mesothelioma by targeting anti-apoptotic pathways

**DOI:** 10.20517/cdr.2019.41

**Published:** 2019-12-19

**Authors:** Marissa Williams, Yuen Yee Cheng, Monica Phimmachanh, Patrick Winata, Nico van Zandwijk, Glen Reid

**Affiliations:** ^1^Asbestos Diseases Research Institute, Sydney NSW2139, Australia.; ^2^Sydney Medical School, The University of Sydney, Sydney NSW2050, Australia.; ^§^Current address: Sydney Local Health District, Concord, Sydney NSW2194, Australia.; ^†^Current address: Department of Pathology, University of Otago, Dunedin 9016, New Zealand.; ^≠^Current address: Garvan Institute of Medical Research, Darlinghurst, Sydney NSW2010, Australia.

**Keywords:** Malignant pleural mesothelioma, miR-15a, miR-16, miR-34a, BCL2, apoptosis

## Abstract

**Aim:** Aberrant microRNA expression is a common event in cancer drug resistance, however its involvement in malignant pleural mesothelioma (MPM) drug resistance is largely unexplored. We aimed to investigate the contribution of microRNAs to the resistance to drugs commonly used in the treatment of MPM.

**Methods:** Drug resistant MPM cell lines were generated by treatment with cisplatin, gemcitabine or vinorelbine. Expression of microRNAs was quantified using RT-qPCR. Apoptosis and drug sensitivity assays were carried out following transfection with microRNA mimics or BCL2 siRNAs combined with drugs.

**Results:** Expression of miR-15a, miR-16 and miR-34a was downregulated in MPM cells with acquired drug resistance. Transfection with miR-15a or miR-16 mimics reversed the resistance to cisplatin, gemcitabine or vinorelbine, whereas miR-34a reversed cisplatin and vinorelbine resistance only. Similarly, in parental cell lines, miR-15a or miR-16 mimics sensitised cells to all drugs, whereas miR-34a increased response to cisplatin and vinorelbine. Increased microRNA expression increased drug-induced apoptosis and caused BCL2 mRNA and protein reduction. RNAi-mediated knockdown of BCL2 partly recapitulated the increase in drug sensitivity in cisplatin and vinorelbine treated cells.

**Conclusion:** Drug-resistant MPM cell lines exhibited reduced expression of tumour suppressor microRNAs. Increasing tumour suppressor of microRNA expression sensitised both drug resistant and parental cell lines to chemotherapeutic agents, in part through targeting of BCL2. Taken together, these data suggest that miR-15a, miR-16 and miR-34a are involved in the acquired and intrinsic drug resistance phenotype of MPM cells.

## Introduction

MPM is an aggressive tumour caused by the inhalation of asbestos fibres^[1]^. The prognosis for MPM is extremely poor, which is mainly attributed to the tumour being inherently chemo-resistant resulting in poor response to available therapies^[2-4]^. Only approximately 40% of patients will respond to the combined cisplatin and pemetrexed treatment regime, which results in a modest median survival of 12 months^[2]^. Further, response to agents routinely used in second- or third-line therapy, including gemcitabine and vinorelbine, is poor with less than 30% of patients demonstrating disease control after 3 months of treatment and median overall survival rates less that 6 months^[5-7]^. The exact mechanisms causing drug resistance in MPM are not completely understood and evidence so far suggests that the mechanisms responsible are likely to be multifaceted^[2]^. Uncovering the molecular pathways driving mechanisms of resistance and how they are regulated is necessary to identify new therapeutic targets and treatment approaches^[4]^.

MicroRNAs (miRNAs) are small non-coding RNAs that post-transcriptionally repress gene expression. MiRNAs are involved in all essential biological pathways, and consequently their dysfunction is often implicated in the development of the hallmarks of cancer^[4]^. Deregulated miRNA expression is widely encountered in tumours^[8]^, making them potential biomarkers and therapeutic targets^[4]^. Generally, miRNA levels are described to be globally downregulated in cancer, and many of these miRNAs exert tumour suppressive function by targeting transcripts involved in tumorigenesis^[8,9]^. Among their many roles in cancer biology, miRNAs have been specifically implicated in tumour drug resistance^[10]^. In an early study, global miRNA expression changes were determined in response to an extensive range of anti-cancer agents in a panel of 60 diverse human cancer cell lines (the NCI-60) and significant correlations between miRNA expression and the potency of the chemical compounds tested were found, suggesting a role for miRNAs in the development of chemo-resistance^[11]^.

Aberrant miRNA expression is a common event in MPM and has been evidenced to contribute to MPM pathogenesis and progression^[9]^. In contrast, investigations into the role of miRNAs in MPM drug resistance are still in their infancy. A recent report showed that re-established miR-31 expression, which is often lost in MPM, was found to reduce cisplatin and carboplatin chemo-sensitivity of MPM cell lines via an unknown mechanism that was linked to reduced intra-nuclear platinum accumulation^[4]^. Previously, we demonstrated the consistent downregulation of the tumour suppressor miR-15/16 family in MPM tumours and cell lines. Re-expression of miR-16 inhibited cell growth *in vitro* and in xenograft bearing nude mice and was shown to sensitise MPM cells to gemcitabine and pemetrexed^[12]^. The restoration of miR-15/16 family expression has been previously implicated in re-establishing chemo-sensitivity in gastric cancer^[10]^, osteosarcoma^[13]^ and breast cancer^[14]^ via modulation of positive regulators in anti-apoptotic pathways.

Additionally, the loss of miR-34 family members is frequently documented in cancer, with enforced expression shown to increase drug response in tumour cells^[15,16]^. Upregulation of miR-34a was shown to attenuate drug resistance to 5-fluorouracil in colon cancer via downregulation of targets in the PI3K/AKT signaling pathway^[17]^ and its restoration elicited increased sensitivity to cisplatin in bladder cancer cell lines by transcriptional inhibition of SIRT-1 and Cdk6^[18]^ . Further, in multi-drug resistant osteosarcoma cells, miR-34b levels were increased with sirolimus treatment, an mTOR inhibitor^[19]^. Elevated miR-34b expression led to increased sensitivity to gemcitabine, doxorubicin, and methotrexate and enhanced drug-induced apoptosis. This was presumably via miR-34b modulation of PAK1 and ABCB1, which are key regulators of cell cycle, multi-drug resistance and apoptosis. The miR-34 family are transcriptional targets of p53, and mediate some downstream p53-functions including regulation of cell proliferation, invasion, and induction of apoptosis^[16,20]^. Epigenetic silencing of members of the miR-34 family, predominantly miR-34b/c, appears to be involved in the tumorigenesis of MPM^[20]^, but its contribution to MPM chemo-resistance, like the miR-15/16 family, is largely unexplored.

As miRNAs have the potential to re-sensitise tumour cells to chemotherapeutic agents, this study aimed to explore their contribution to drug resistance in MPM. This investigation demonstrates that acquired drug resistance in MPM is associated with suppression of miR-15a, miR-16 and miR-34a. In particular, miR-15a and miR-16 were demonstrated to play a role in the development of acquired and intrinsic resistance in MPM cancer cells by modulating drug-induced apoptosis, at least in part via regulation of B-cell lymphoma 2 (BCL2) protein expression.

## Methods

### Cell lines and cultures

The human MPM cell line MSTO-211H was obtained from the ATCC. The parental cells (MSTO-Par) and sub-lines resistant to cisplatin (MSTO-CisR), gemcitabine (MSTO-GemR) and vinorelbine (MSTO-VinoR) were cultured in RPMI-1640 medium supplemented with 10% FCS (both Thermo Fisher Scientific, Carlsbad, CA, USA) with 5% CO_2_ at 37 °C. Drug-resistant cell lines (MSTO-CisR, MSTO-GemR and MSTO-VinoR) were established by standard cyclic treatment with the IC_50_ of cisplatin, gemcitabine and vinorelbine (all drugs purchased from Sigma Aldrich, St. Louis, MO), respectively. Drug concentrations were increased incrementally until a resistance of 2 to 5-fold was reached, after which they were maintained by treatment with constant concentrations of drug (10 µmol/L for cisplatin and 20 nmol/L for gemcitabine and vinorelbine) from 24 h following passaging. Resistance gradually increased over a period of 3-4 months to the final resistance levels described in Results. Resistance was regularly confirmed by comparison of drug IC_50_ values in the drug resistant cell lines and parental line.

### Transfection with miRNA mimics or siRNAs combined with drug treatment

Restoration of miRNA expression was performed using miRNA mimics corresponding to mature miRNA sequences and knockdown of BCL2 was carried out using two siRNA sequences designed in our laboratory. All miRNA mimics, BCL2 siRNAs, and controls were purchased from GenePharma (Shanghai, China) and are listed in Supplementary Table 1. For cell proliferation assays, cells were transfected in 96-well plates with mimics/siRNAs or controls at a final concentration of 0.5 or 1 nmol/L, as previously described^[12]^. At 24 h post transfection, cells were treated with 2-fold serial dilutions of cisplatin, gemcitabine or vinorelbine at the concentrations indicated. Cells were then incubated for a further 96 h after which medium was removed and plates were frozen at -80 °C until they were assayed for cell proliferation. For RT-qPCR, Western blotting and analysis of apoptosis, 1.5 × 10^5^ cells per well in 6-well plates were reverse transfected with mimics and siRNAs at the final concentrations indicated.

### Real time RT-qPCR for miRNA and mRNA quantification

Total RNA was extracted from control and miRNA mimic transfected cell lines 24 h post transfection using Trizol (Thermo Fisher scientific) according to the manufacturer’s instructions. RNA was quantified using a nanophotometer (Implen, Munich, Germany) with readings at 260 nmol/L and 280 nmol/L, after which it was stored at -80 °C before subsequent analysis. For miRNA quantification, reverse transcription was carried out as described previously^[21]^. Briefly, 100 ng of total RNA was reverse transcribed with a 4 µL of an equimolar mixture of pooled 5X TaqMan stem-loop primer assays (Thermo Fisher scientific; TaqMan IDs listed in Supplementary Table 1) using the MicroRNA Reverse Transcription Kit (Thermo Fisher scientific) in a final reaction volume of 10 µL, with the following reaction conditions: 30 min at 16 °C, 30 min at 42 °C and 5 min at 85 °C. The resultant cDNA was then diluted with 58.7 µL of water after which 2.25 µL was analysed for miRNA expression using the KAPA FAST Probe mastermix (KAPA Biosystems, Wilmington, MA) according to manufacturer’s instructions. MicroRNA-specific hydrolysis probes (Thermo Fisher Scientific; Supplementary Table 1) were used to amplify miRNA expression on a Viia 7 real time machine (Thermo Fisher Scientific) with the cycling conditions: 20 s initial denaturing at 95 °C, followed by 40 cycles of 1 s at 95 °C and 20 s amplification at 60 °C. The change in miRNA expression in MSTO drug resistant cell lines relative to the parental control cell line (MSTO-Par) was calculated using the 2^-ΔΔCT^ method^[22]^, where ΔΔCt = ΔCt MSTO-CisR/GemR/VinoR -ΔCt MSTO parental and ΔCt = Ct miRNA-Ct RNU6.

To measure mRNA expression, 1 µg total RNA was reverse transcribed using the Promega (Madison, WI) MMLV Reverse Transcription kit according to manufacturer’s instructions. First, RNA was incubated at 72 °C for 5min with a combination of Oligo(dT)_15-18_ primers (12.5 µmol/L), 2.5 mmol/L dNTP-mix and random hexamers (625 nmol/L) for primer annealing. 1X MMLV reaction buffer and 100 units of MMLV Reverse Transcriptase were then added to the RNA mixture, under the following reaction conditions: 42 °C for 60 min and 94 °C for 10 min. The resultant cDNA was then diluted to a final concentration of 40 ng/µL and added as the template for qPCR analysis with forward and reverse primers [Supplementary Table 1] and KAPA SYBR Fast qPCR master mix (Kapa Biosystems). Reactions were performed on the Viia7 real-time machine (Thermo Fisher Scientific) and had an initial enzyme inactivation step at 95 °C for 10 min followed by 40 cycles of 95 °C for 15 s and 55 °C for 30 s. Relative expression levels were calculated using the 2^-ΔΔCT^ method where expression was normalised to the 18S ribosomal RNA as reference.

### Cell proliferation assay

Cell proliferation was measured using a SYBR Green-based cell assay, as described previously^[23]^. To each well of the 96 well plate, 150 µL of hypotonic lysis buffer (10 mmol/L Tris HCl (pH 8), 5 mmol/L EDTA, 0.1% Triton X-100) containing 1:8000 SYBR green I Nucleic Acid Gel Stain (Thermo Fisher Scientific) was added. Plates were incubated overnight in the dark at 4 °C after which Relative Fluorescent Units (RFU) for each well was quantified by fluorimetry, measured using a FLUOstar Optima microplate reader (BMG LabTech, Ortenberg, Germany) set to 485 nm excitation and 535 nm emission. Fluorescence intensity in drug and mimic/siRNA-transfected cells was normalised to that of mimic or siRNA transfected cells only, to eliminate any toxicity induced by mimic/siRNA treatment alone.

### Apoptosis assay

The Tali Image-Based Cytometer (Thermo Fisher Scientific) was used to measure levels of apoptosis, necrosis, and death in transfected cells after treatment with cisplatin, gemcitabine and vinorelbine, using the Tali Apoptosis Kit (Thermo Fisher Scientific). 24 h following transfection with miRNA mimics or controls in 6-well plates, drugs were added to cells at a dose between the IC_50_ values of parental and resistant lines for each respective agent and varied from 50 nmol/L to 3 µmol/L. Following 48 h of drug treatment, cells were resuspended in 100 mL of apoptosis buffer with 5 mL of Annexin V and incubated for 20 min in the dark at room temperature. Samples were then centrifuged, and cell pellets resuspended in 100 µL of apoptosis buffer containing 1 µL propidium iodide (PI). After 5 min in the dark at room temperature, cells were analysed on the Tali Image-Based Cytometer. Cells were considered to be apoptotic when stained with annexin, dead when stained with PI and late apoptotic/necrotic when stained with both dyes. Each sample was measured across 18-fields of view where total cell numbers for each type of fluorescence was determined with digital image-based counting and fluorescence detection algorithms.

### Western blot analysis

Cells were reverse transfected with miRNA mimics or BCL2 siRNAs (1 nmol/L) in 6-well plates and 48 h post transfection BCL2 protein expression was determined using Western blotting as described previously^[12]^. Briefly, 30 µg of denatured protein was separated on a 10% polyacrylamide gel and then transferred to polyvinylidene fluoride membranes (Bio-Rad), blocked and probed with a BCL2-specific rabbit-monoclonal antibody (D55G8; Cell Signalling Technology, Danvers, MA) diluted 1:500 in blocking buffer. Anti-rabbit peroxidase-conjugated secondary antibody (Ab6721; Abcam, Cambridge, United Kingdom; diluted 1:2500) was used for detection via chemiluminescence (Clarity Western ECL substrate [Bio-Rad]) using the ChemiDoc MP system (Bio-Rad). For determination of loading control expression, membranes were stripped and re-probed with a Beta-actin (b-actin)-specific antibody (AC74; Sigma-Aldrich). Quantitative analysis of protein bands was accomplished by densitometry using the Image J 1.48 software. BCL2 protein band values were taken as a percentage of values calculated for b-actin and then normalised to control transfected cells.

### Luciferase reporter assays

A 278-bp fragment of the BCL2 3’UTR region containing binding sites for miR-15a/miR-16 (corresponding to nucleotides 3605-3882 in the RefSeq entry for *BCL2*) was identified and cloned from total RNA isolated from MSTO cells as described previously^[24]^. The resultant amplicon was subcloned into the pSiCheck2 plasmid (Promega) after confirmation of sequence identity by Sanger sequencing (Ramaciotti Centre, UNSW, Sydney). Cells (500,000) were transfected with 1 µg of the resultant reporter construct, together with miRNA mimics and controls in 6-well plates and after 48 h post-transfection, the dual luciferase assay (Promega) was carried out according to manufacturer’s instructions using a FLUOstar Optima plate reader. An average of the RFUs for luciferase and renilla measurements were taken and a ratio was taken of renilla/luciferase after which fold-changes were determined for transfected cells vs scrambled control.

### Statistical analysis

Differences in miRNA and mRNA expression levels in cell lines were analysed with two-tailed independent samples *t*-test. Mann-Whitney-*U*-test was used to assess differences in cell proliferation. All analyses were performed using SPSS Statistics version 25 (IBM Corp., Armonk, NY) and a *P*-value of ≤ 0.05 was considered statistically significant. IC_50_ values were determined using the forecast function in excel (Version 16.13.1).

## Results

### Tumour suppressor miRNAs are expressed differentially in drug resistant cell lines compared to their parental counterpart

**Figure 1:**
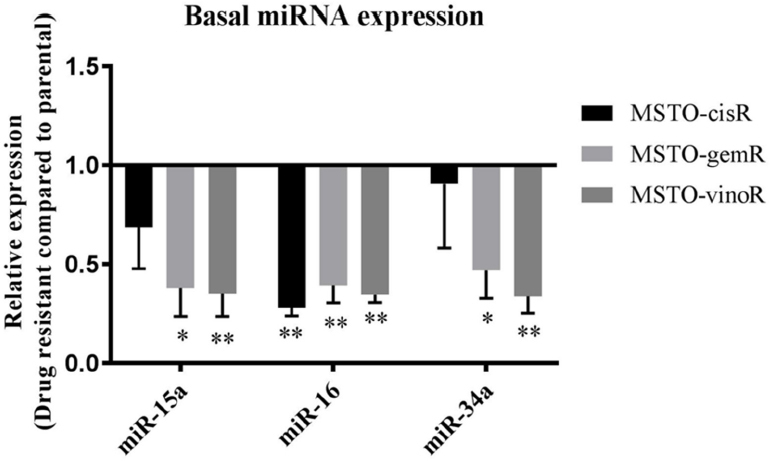
Tumour suppressor miRNAs are reduced in drug resistant cell lines compared to the parental cell line. Levels of mature miRNAs were measured in MPM drug resistant cell lines and related to levels in the parental MSTO cell line using RT-qPCR. miR-15a, miR-16 and miR-34a levels were reduced in MSTO-CisR, MSTO-GemR and MSTO-VinoR compared to MSTO-Par. Expression was normalised to RNU6B and is the mean from 3 independent experiments ± SD. *P ≤ 0.05, **P ≤ 0.01

To determine whether previously identified downregulated tumour suppressor miRNAs are involved in MPM drug resistance, we measured levels of miRNAs in MPM cell lines with acquired drug resistance to cisplatin (MSTO-CisR), vinorelbine (MSTO-VinoR) and gemcitabine (MSTO-GemR) (see Methods). Drug resistant lines were 2.5-fold (MSTO-CisR), 2-fold (MSTO-VinoR) and 6-fold (MSTO-GemR) more resistant than the parental line [Supplementary Figure 1]. Levels of tumour suppressor miRNAs were measured in parental and drug resistant cell lines using RT-qPCR. Levels of miR-15a, miR-16 and miR-34a are known to be downregulated in MPM tumour samples and cell lines compared to normal controls^[12,25]^ and these were found to be further reduced in all 3 drug-resistant lines [Figure 1]. Basal levels of miR-15a and miR-34a were reduced most prominently in MSTO-GemR (2.6-fold and 2.1-fold respectively, Figure 1) and MSTO-VinoR (2.8-fold and 3-fold respectively, Figure 1), while miR-16 levels were found to be consistently low in all drug resistant cell lines compared to the parental cell line. MiR-34b levels were undetectable in all cell lines including the MSTO parental cell line (data not shown). This absence of expression can be attributed to the commonly encountered methylation of the miR-34b/c region in MPM^[25]^.

### Restoring expression of miR-15, miR-16 or miR-34 sensitises cells to drug induced cytotoxicity

**Figure 2:**
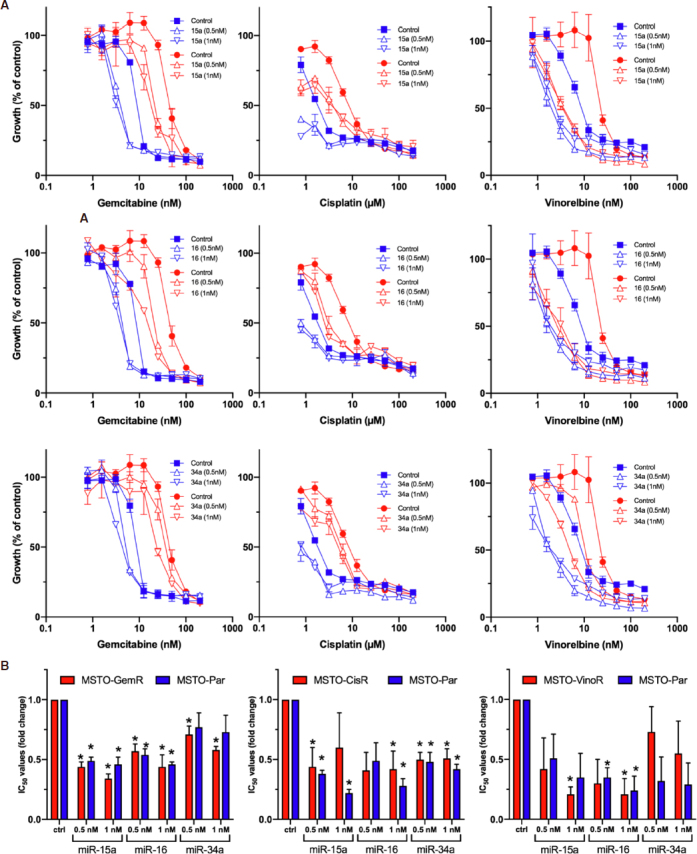
MiRNA mimic-induced sensitisation to chemotherapeutics in MPM cell lines. A: Transfection with mimics for miR-15a, miR-16 and miR-34a reverses drug resistance in parental and drug resistant cell lines. Cells were transfected with miRNA mimics (at concentrations of 0.5 nmol/L and 1 nmol/L) together with a control (1 nmol/L). After 24 h, MSTO parental cells (blue) together with MSTO-GemR, MSTO-CisR and MSTO-VinoR drug-resistant cell lines (red), were treated with 2-fold serial dilutions of gemcitabine, cisplatin and vinorelbine respectively for 96 h after which cell proliferation was assayed. Drug and mimic treated cells were normalised to mimic treatment alone (mimic or control transfected = 100 %). Data are the mean ± SD of duplicate measurements and are representative of 3 experiments producing similar results; B: Drug IC_50_ ratios with miRNA mimic transfection in MPM cell lines. The average reductions of chemotherapeutic IC_50_ values induced by miRNA restoration, across 3 replicate experiments, were depicted as a ratio of the IC_50_ values from mimic transfected cells compared to those transfected with a control mimic. Data is the average of 3 replicate experiments ± SEM. *P < 0.05 (two-tailed independent samples *t*-test)

We next investigated the functional impact of reduced tumour suppressor microRNA expression to the development of acquired drug resistance in MPM. We transfected MPM cells with mimics corresponding to miR-15a, miR-16 or miR-34a and then treated with drug. Cell proliferation assays showed shifts in drug sensitivity in a dose-dependent manner in microRNA mimic transfected cells in both resistant and parental cells. All 3 miRNAs reduced the resistance to all 3 drugs, as well as increasing the sensitivity of the parental cells [Figure 2]. In the case of cisplatin and vinorelbine, the resistance of the drug-selected lines was completely reversed, and the sensitivity of both parental and resistant lines were reduced to similar IC_50_ values. In contrast, gemcitabine resistance was only partly reversed by transfection with miRNAs, and the IC_50_ values remained slightly higher than the parental line.

### Restoring miR-15a, miR-16 and miR-34a induced apoptosis in drug-resistant cell lines

**Figure 3:**
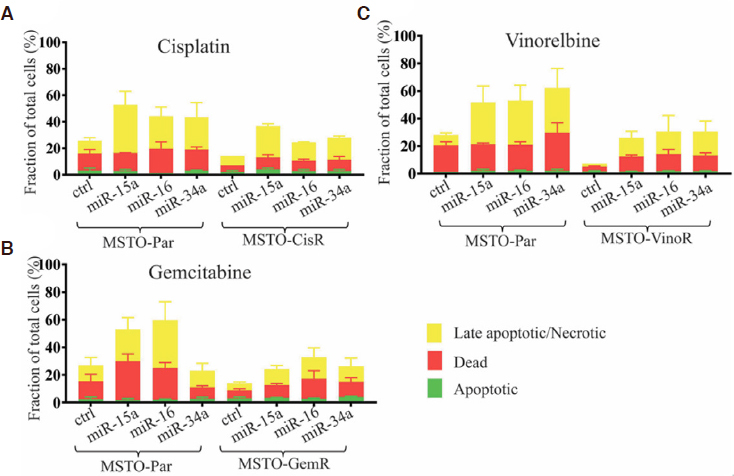
Transfection with miR-15a, miR-16, and to a lesser extent miR-34a increases levels of drug-induced apoptosis. MPM cells were transfected with miRNA mimics or control (0.5 nmol/L) and were then treated with (A) cisplatin, (B) gemcitabine or (C) vinorelbine 24 h later (3 μmol/L-50 nmol/L). Tali Apoptosis assays demonstrated changes in cellular levels of apoptosis, late apoptosis/necrosis and death 72 h post-transfection. Data are the mean of 3 independent experiments ± SEM

The development of drug resistance is often attributed to the escape of drug induced apoptosis in cancer cells^[10]^. Considering that the miR-15/16 family and miR-34a have targets involved in apoptosis we hypothesised that their restoration may reinstate drug sensitivity by influencing apoptotic pathways. All drug resistant cells displayed consistently lower levels of drug-induced apoptosis and death compared to the parental counterpart, confirming their resistance to the effects of chemotherapeutic drugs [Figure 3]. Transfection with mimics of miR-15a or miR-16 increased drug sensitivity and induced marked increases in cellular levels of late apoptosis in MPM cell lines in response to treatment with cisplatin [Figure 3A] or gemcitabine [Figure 3B]. Re-expression of miR-34a also increased apoptosis in response to drug cisplatin or gemcitabine, albeit to a lesser extent [Figure 3A and B]. Late apoptotic cell death markers were increased most prominently in vinorelbine-treated cells, rising approximately 7-fold following transfection with miR-15a, miR-16 or miR-34a mimics in MSTO-VinoR cells and 4-fold in parental cells [Figure 3C]. Collectively this data demonstrates that restoration of miR-15a, miR-16, and to a lesser extent miR-34a expression, universally increases cellular levels of late apoptosis in response to a panel of clinically relevant chemotherapeutic drugs.

### Transfection with miR-15a, miR-16 or miR-34a mimics reduces BCL2 mRNA and protein levels

**Figure 4:**
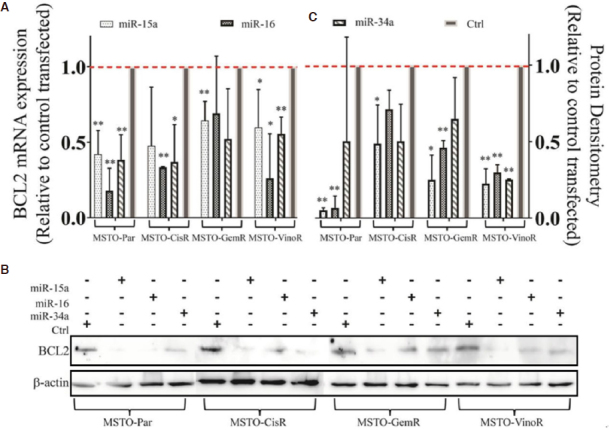
BCL2 expression is reduced following transfection with miRNA mimics. miR-15a, miR-16 and miR-34a are predicted to bind to the 3’UTR region of the BCL2 mRNA and negatively regulate its expression. (A) Transfection with mimics (1 nmol/L) for miR-15a, miR-16 and miR-34a were shown to reduce BCL2 mRNA levels after 24 h in MSTO-Par and resistant cell lines compared to transfection with a control as demonstrated by RT-qPCR. Data are normalised to 18 s and are a mean of 3 independent experiments ± SD. *P ≤ 0.01. (B) Changes in BCL2 expression following 1 nmol/L mimic transfection (48 h) are reiterated on a protein level as shown by western blotting. Protein densitometry confirms this reduction (C) and data are a mean of 3 independent experiments ± SD. *P ≤ 0.05, **P ≤ 0.01

A major mechanism contributing to cancer drug resistance is the avoidance of drug-induced apoptosis, which has been shown to be attributed to the overexpression of various anti-apoptotic proteins such as BCL2^[26]^. BCL2 is a direct target for post-transcriptional repression by the miR-15/16 family^[27]^ and miR-34a^[28]^, and coupled with its well-established role in chemo-resistance, we hypothesised that the observed miRNA-dependent sensitisation to drug-induced apoptosis in the resistant cell lines might be explained by miRNA regulation of anti-apoptotic BCL2. To investigate this possibility, BCL2 expression was determined in MPM resistant and parental cell lines following transfection with miR-15a, miR-16, or miR-34a mimics. All mimics reduced BCL2 mRNA and protein levels compared to control transfected cells in both parental and resistant cell lines [Figure 4]. MiR-15a mimic transfection is shown to almost completely abolish BCL2 expression in all cell lines while transfection with the miR-16 mimic reduced expression by more than 50% in the majority of cell lines which is comparable to reductions observed using siRNA induced knockdown [Supplementary Figure 2]. We further assessed the direct association of miR-15a and miR-16 with the BCL2 mRNA using luciferase assays, and found that both interact with the 3’UTR region of the BCL2 mRNA [Supplementary Figure 3], confirming their direct regulation of this protein in MPM.

### Downregulation of BCL2 expression is not as effective at improving chemo-sensitivity as increasing microRNA expression

**Figure 5:**
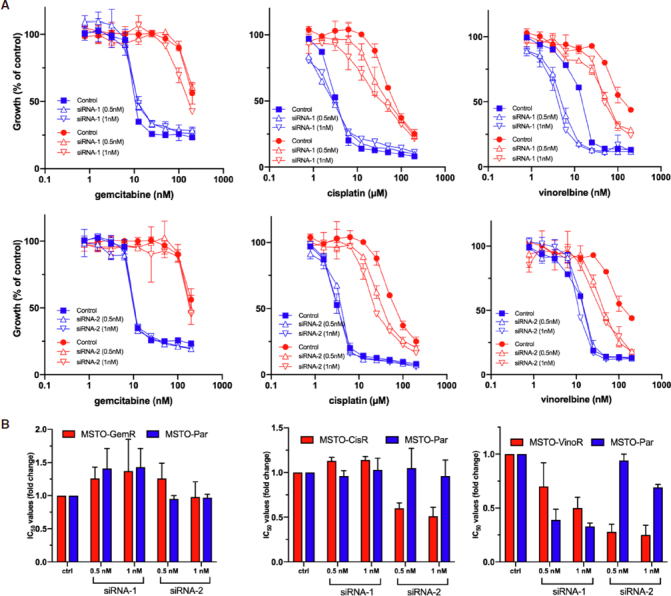
Drug sensitivities following BCL2 knockdown. A: Cells were transfected with BCL2 siRNAs (siRNA-1 and siRNA-2 at 0.5 nmol/L and 1 nmol/L) or a control (1 nmol/L). After 24 h MSTO parental (blue) and MSTO-GemR, MSTO-CisR and MSTO-VinoR resistant cell lines (red), were treated with 2-fold serial dilutions of gemcitabine, cisplatin or vinorelbine respectively. Cells were assayed for proliferation 96 h following drug treatment. Drug and siRNA treated cells were normalised to siRNA treatment alone (siRNA or control transfected = 100%). Data are mean ± SD of duplicate measurements and are representative of 3 experiments producing similar results; B: The average changes of chemotherapeutic IC_50_ values induced by BCL2 siRNA transfection, across 3 replicate experiments, were depicted as a ratio of the IC_50_ values from siRNA transfected cells compared to those transfected with a control siRNA. Data is the average of 3 replicate experiments ± SEM. *P < 0.05 (two-tailed independent samples *t*-test)

To further investigate the idea that miR-15, miR-16 and miR-34a modulate drug resistance via targeting the anti-apoptotic protein BCL2, its expression was reduced with specific siRNAs and the subsequent effect on cell growth was evaluated. BCL2 downregulation caused only a modest sensitisation to vinorelbine and cisplatin treatment, whereas the effects of gemcitabine treatment was unchanged [Figure 5; Table 2]. In contrast to the effects of microRNA mimic transfections, the BCL2 siRNA-mediated sensitisation to cisplatin and vinorelbine was only apparent in the resistant cell line and not in the parental line [Figure 5; Table 2]. Similar to trends observed with miRNA mimic transfection, vinorelbine sensitivity was most altered of all the drugs tested in response to direct BCL2 repression in both MSTO-VinoR and MSTO-Par cell lines [Figure 5]. Collectively, this data demonstrates that despite being a confirmed target of both miR-15a, miR-16 and miR-34a, direct downregulation of BCL2 did not confer the same extent of drug sensitisation as miRNA restoration.

## Discussion

MPM displays remarkable resistance to current clinical treatment regimens and new approaches are urgently needed^[29]^. MiRNAs are known to contribute to drug sensitivities of cancer cells and we previously demonstrated the ability of miR-16 mimics to sensitise MPM cells to gemcitabine and pemetrexed^[12]^. In an attempt to further characterise the role of miRNAs in MPM drug resistance, we established drug resistant models by periodic chemotherapeutic treatment of the MSTO cell line. Our study demonstrated that the co-transcribed miRNAs miR-16 and miR-15a, together with miR-34a, were consistently downregulated in MPM cell lines with acquired resistance to cisplatin, gemcitabine and vinorelbine. As these miRNAs are frequently associated with cancer it is likely that they regulate cellular phenotypes involved in carcinogenesis and drug resistance.

Tumour suppressor miRNAs have diverse roles in the biology underlying chemo-resistance^[30,31]^. MiRNAs have been shown to alter the activity of multidrug-resistant (MDR) transporters in cancer. Specifically, miR-138 re-expression was responsible for the downregulation of ABCB1 (Pgp/MDR1), subsequently leading to increased apoptosis and reversal of Adriamycin resistance in MDR leukemia cells^[32]^. An alternate route of miRNA directed chemo-sensitivity is via the increase of tumour cell autophagy, where miR-15a/16 was shown to target components of the mTORC1 complex to enhance camptothecin-induced autophagy^[33]^. MiRNAs are also known to regulate apoptotic machinery; let-7 expression increased apoptosis in response to doxorubicin treatment by targeting caspase-3 and subsequent drug sensitivity in a panel of cancer cell lines^[34]^. As well as influencing autophagy to modulate drug resistance, miR-15/16 has also been implicated in the cellular induction of apoptosis by targeting BCL2^[10,27,35]^. Similarly, miR-34a, another miRNA found to be differentially expressed in drug resistant cells in this study, was also demonstrated to promote apoptosis and enhance tumour cell response via pathways involving BCL2 repression^[28,36,37]^. To the best of our knowledge, however, no reports have detailed whether the described anti-apoptotic mechanisms of sensitisation mediated by miR-15a, miR-16 and miR-34a are explicitly involved in MPM drug resistance.

Apoptosis is the process of programmed cell death and represents a major response to DNA damage induced by anti-cancer drugs^[28,38]^. Cisplatin in combination with other drugs remains the main treatment strategy for mesothelioma, and its anticancer activity relies on the activation of apoptosis to execute cell death^[3]^. A defective apoptosis pathway is a major mechanism contributing to drug resistance in cancer^[38,39]^ and multiple investigations have demonstrated a role for miRNAs in modulating this mechanism of cancer drug resistance^[26,38]^. Exogenous upregulation of miR-15/16 was shown to reverse the multidrug resistance of a gastric cancer cell line to a panel of anticancer drugs via direct regulation of the anti-apoptotic BCL2 protein^[10]^ and to induce the intrinsic apoptosis pathway via negative regulation of BCL2 expression in CLL^[27]^. In breast cancer, miR-15a/16-1 was downregulated in tamoxifen-resistant tumours due to overexpression of the human epidermal growth factor (HER2). Re-expression of miR-15a/16-1 led to the suppression of BCL2 which sensitised cells to chemotherapy^[35]^. Similarly, miR-34a was shown to repress BCL2 expression, leading to sensitisation to sorafenib-induced apoptosis in hepatocellular carcinoma^[28]^ and increased levels of apoptosis via the mitochondrial death cascade in non-small cell lung carcinoma (NSCLC) cells^[40]^. In our investigation, we found a consistent downregulation of miR-15a, miR-16 and miR-34a in all drug-resistant cell lines. This change was of functional significance as restoration of their expression led to reduced resistance of cells to chemotherapeutic drugs.

Previously, miR-15a, miR-16 and miR-34a have been shown to directly interact with the 3’UTR region of the BCL2 mRNA and repress its expression^[27,28]^; our work here confirms this regulation is also apparent in MPM and suggests the likely involvement of BCL2 in modulating the susceptibility of MPM cells to drug-induced apoptosis. To further understand this proposed mechanism of drug sensitisation, we reduced BCL2 levels via siRNA-mediated knockdown. Although this increased vinorelbine and cisplatin sensitivity in MSTO-VinoR and MSTO-CisR cell lines respectively, the extent of chemo-sensitisation was significantly less than that elicited by miRNA mimics. This finding is not surprising considering miRNAs target numerous genes implicated in cancer drug resistance^[39]^, making it likely that multiple additional pathways involved in MPM drug resistance are targeted by miR-15a, miR-16 and miR-34a. This is consistent with a study in bladder cancer, where knockdown of Cdk6 and SIRT-1 (direct targets of miR-34a) was not as effective reversing cisplatin resistance as miR-34a restoration^[18]^. Further, we found that the parental MSTO cell line did not display shifts in cisplatin and gemcitabine sensitivity with BCL2 siRNA transfection, suggesting that miRNA mediated reversal of BCL2-dependent apoptosis evasion is exclusive to cells with acquired drug resistance. Alternative anti-apoptotic targets of miR-15a, miR-16 and miR-34 have been implicated in cancer chemo-resistance and may also have roles in MPM. For example, expression of miR-16 was found to be downregulated in paclitaxel-resistant breast cancer cell lines, and exogenous overexpression of miR-16 sensitised breast cancer cells to Paclitaxel induced apoptosis and cytotoxicity via direct modulation of IκB kinase β (IKBKB)^[14]^. In a further study, miR-15a and miR-16 expression was significantly downregulated in tamoxifen-resistant breast cancer cells and were able to enhance apoptosis via regulation of both Cyclin E1 and BCL2 expression^[41]^. Upregulation of miR-16 also elevated levels of apoptosis in multiple cancer cell lines by directly repressing the expression of the FEAT oncogene^[42]^. Additionally, miR-34a downregulation was recently associated with poor overall survival in breast cancer patients and multi-drug resistance in breast cancer cell lines. Ectopic upregulation of miR-34a expression led to partial MDR reversal, reduction of target genes Ccnd1, Notch1 and BCL2 expression and increased apoptotic rates in MDR breast cancer cells^[43]^. Paclitaxel resistance has also been associated with the loss of miR-34a in prostate cancer. Micellar delivery of Rubone, a small molecule enhancer of miR-34a expression, led to induction of apoptosis, repression of the miR-34a targets SIRT-1, E-cadherin and Cyclin D and increased paclitaxel sensitivity^[44]^.

It is worth noting that siRNA-mediated BCL2 knockdown did not modulate the cytotoxicity of gemcitabine. This data suggests that it is possible that miRNA upregulation triggers gemcitabine induced apoptosis through a pathway where BCL2 is not necessarily involved. In support of this, gemcitabine was previously demonstrated to induce apoptosis via the Fas/FasL pathway in lung cancer cells^[45]^. If a similar phenomenon is present in MPM cells then factors that regulate the intrinsic mitochondrial apoptotic pathway - such as BCL2 - will have negligible effects on the cell response to gemcitabine-induced apoptosis^[10]^. The combined data here suggest that miR-15a/16-1 and miR-34a are likely to target multiple factors within apoptotic pathways to induce drug-senstitisation in MPM cells.

Finally, it was interesting to see that cells were most responsive to vinorelbine sensitisation following both miRNA restoration and direct BCL2 repression, and this was apparent in both the MSTO-VinoR and MSTO-Par cells. NSCLC cells have previously been demonstrated to be susceptible to increased vinorelbine sensitisation by curcumin addition; cells were sensitised to vinorelbine-induced apoptosis by modulation of components of the mitochondrial pathway including BCL2, Bax, Bcl-xs, and Bcl-X, thereby releasing apoptogenic cytochrome c^[46]^. Reversal of vinorelbine resistance may follow a similar mechanism in MPM and would explain the enhanced levels of drug-sensitisation with BCL2 modulation both indirectly via miRNA restoration and directly with BCL2 siRNA knockdown.

Our study may have implications for improving the poor prognosis of MPM, which is attributed to the impaired response to platinum-based chemotherapeutics applied as first-line treatment options for MPM patients. Efficacy of current chemotherapies used in MPM are often impeded by the development of drug-resistant phenotypes and new options are urgently required^[4]^. MiRNAs involved in drug-resistance, such as miR-15a, miR-16 and miR-34a, represent attractive synergistic therapeutic targets to improve chemotherapy based on their ability to regulate pathways through the control of multiple genes; this global modulation is superior to therapeutics targeting single proteins. Recently, the restoration of miR-16 activity using a miR-16-based mimic delivered by bacterially derived, EGFR-targeted, minicells was investigated in a phase 1 clinical trial, and exhibited early signs of activity in MPM patients^[47]^. The drug sensitisation capabilities of miR-16 restoration demonstrated in the current study *in vitro*, together with its potential as a targeted therapeutic agent, suggests that a combination of miR-16 replacement with chemotherapy may be a worthwhile approach for MPM patients.

In summary, we report here that tumour suppressor miRNAs in MPM are capable of sensitising MPM cell lines to clinically relevant chemotherapeutic drugs. MiR-15a and miR-16 significantly increased the sensitivity of MPM cells to 3 clinically used drugs. Although this was in part due to BCL2 modulation, BCL2 knockdown did not fully recapitulate the global drug sensitisation demonstrated by miRNA restoration, meaning it is highly likely that other targets are involved. Our findings provide insights into the miRNA-regulated mechanisms underlying MPM drug resistance and may provide the foundations for future development of utilising miRNAs to improve chemotherapy response.

## Declarations

### Copyright

© The Author(s) 2022.
