# Supplementary material for: Tumour suppressor microRNAs contribute to drug resistance in malignant pleural mesothelioma by targeting anti-apoptotic pathways
Source: Cancer Drug Resist. 2019 Dec 19;2(4):1193–206. doi: 10.20517/cdr.2019.41 (PMC9019216; doi:10.20517/cdr.2019.41)
Supplement: Supplementary file 1 [file cdr-2-1193-SupplementaryMaterials.pdf]

DOI: 10.20517/cdr.xxx.xx

Cancer
Drug Resistance

Supplementary Table 1. Assays, primers and miRNA mimic/siRNA sequences

TaqMan assays		
MicroRNA	TaqMan Assay ID	
miR-15a-5p	000389	
miR-16-5p	000391	
miR-34a-5p	000426	
miR-34b-3p	000427	
RNU6B	001973	
Primers for mRNA expression		
Target	Forward primer (5'-3')	Reverse primer (5'-3')
BCL2	ACAGAGGATCATGCTGTAC	TTATTTCATGAGGCACGTT
18S	GCCGCTAGAGGTGAAATTC	
Primers for reporter assay		
Insert region	Forward primer (5'-3')	Reverse primer (5'-3')
BCL2 3'UTR	ATCGCTCGAGGTATGAAGC	ATCGGCGGCCGCCAACAA
MicroRNA mimics		
MicroRNA	Antisense strand (5'-3')	Sense strand (5'-3')
miR-15a-5p	UAGCAGCACAUAAUGGUU	CAAACCAUUAUGUGCUGC
miR-16-5p	UAGCAGCACGUAAAUAUU	CCAAUAUUUACGUGCUGC
miR-34a-5p	UGGCAGUGUCULIAGCUG	AACCAGCUAAGACACUGC
siRNAs		
Target	Antisense strand (5'-3')	Sense strand (5'-3')
Control	AAGCAACUUGGUAAGACU	CCACACGAGUCUUACCAA
BCL2 siRNA-1	GAGAUAGUGAUGAAGUA	AAUGGAUGUACUUCAUCA
BCL2 siRNA-2	GCCCCAGCAUGCGGCCUC	AAUCAAACAGAGGCCGCA

MiRNA TaqMan IDs are listed together with primers for mRNA expression, reporter assay cloning and miRNA mimic/siRNA sequences

© The Author(s) 2018. Open Access This article is licensed under a Creative Commons Attribution 4.0 International License (https://creativecommons.org/licenses/by/4.0/), which permits unrestricted use, sharing, adaptation, distribution and reproduction in any medium or format, for any purpose, even commercially, as long as you give appropriate credit to the original author(s) and the source, provide a link to the Creative Commons license, and indicate if changes were made.

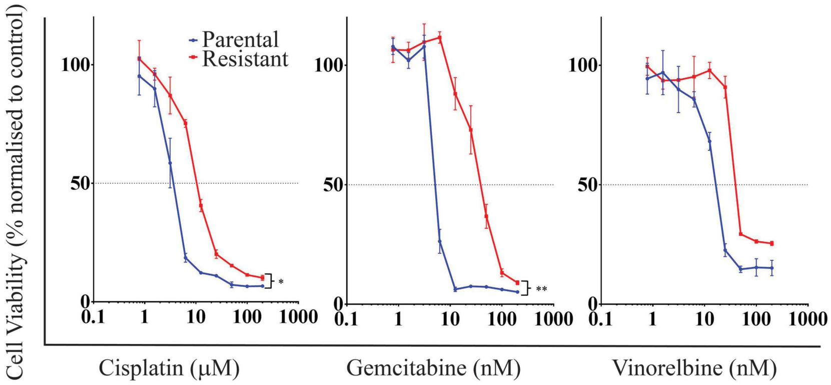

Supplementary Figure 1. Basal drug sensitivities in MSTO parental and drug resistant cell lines. MSTO parental and drug resistant cells (MSTO-CisR, MSTO-GemR, MSTO-VinoR) were treated with 2-fold serial dilutions with the indicated start concentrations of cisplatin, gemcitabine and vinorelbine respectively. Cells were assayed for proliferation 96 h following drug treatment. Drug treated cells were normalised to the values of untreated cells (untreated = 100%). Data are mean ± SD of duplicate measurements and are representative of > 3 experiments producing similar results. $^*P \leq 0.05$

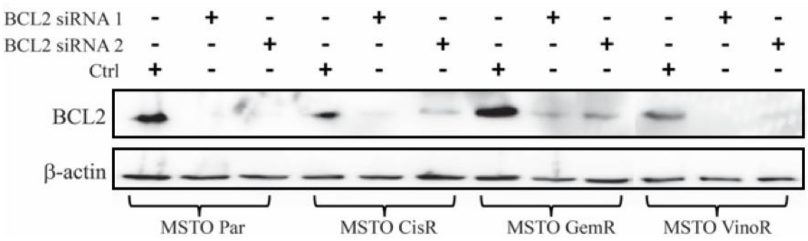

Supplementary Figure 2. Confirmation of BCL2 reduction with BCL2 siRNA transfection. MSTO-Par, MSTO-CisR, MSTO-GemR and MSTO-VinoR were transfected with 1nM of BCL2 siRNAs and after 48 h BCL2 protein expression was measured using western blotting. β-actin was included as a control for equal loading

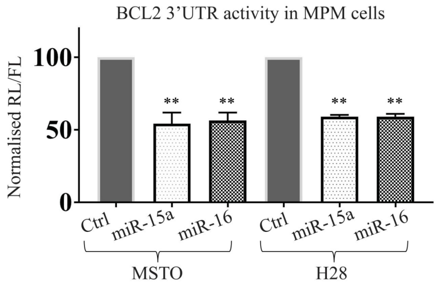

Supplementary Figure 3. Direct regulation of the BCL2 mRNA by miR-15a/16-1. miR-15a and miR-16 reduce luciferase activity of a reporter containing the 3'UTR region of BCL2 mRNA in MSTO-Par and an additional MPM cell line H28. Data is the average of 3 replicate experiments ± SD. **P* ≤ 0.05, ***P* ≤ 0.01
